# Transcriptomic suppression of immune and ECM stability in skeletal muscle of patients with chronic kidney disease

**DOI:** 10.1371/journal.pone.0328947

**Published:** 2026-02-24

**Authors:** Luke A. Baker, Nicholas Eastley, Robert U. Ashford, Matthew Denniff, Matthew Graham-Brown, Emma L. Watson

**Affiliations:** 1 Division of Respiratory Sciences, College of Life Sciences, University of Leicester, Leicester, United Kingdom; 2 NIHR Leicester Biomedical Research Centre (BRC), University of Leicester, Leicester, United Kingdom; 3 Leicester Orthopaedics, University Hospitals of Leicester, Leicester, United Kingdom; 4 Division of Cancer Sciences, College of Life Sciences, University of Leicester, Leicester, United Kingdom; 5 Division of Cardiovascular Sciences, University of Leicester, Leicester, United Kingdom; Versiti Blood Research Institute, UNITED STATES OF AMERICA

## Abstract

**Background:**

Chronic kidney disease (CKD) is a growing public health emergency with a global prevalence of approximately 14%. Sarcopenia is a common complication of CKD contributing to functional decline and poor outcomes. However, the molecular mechanisms driving muscle wasting in CKD remain incompletely understood. This study aimed to characterise the transcriptomic profile in individuals with CKD compared to healthy control counterparts, to identify key pathways implicated in muscle dysfunction.

**Methods:**

Vastus lateralis muscle biopsy samples were obtained from n = 10 people with CKD and n = 9 healthy controls matched for age, sex, ethnicity and physical activity. Bulk RNA sequencing was performed on all samples. Differential gene expression was assessed using DESeq2 and pathway enrichments analyses were conducted using Gene Ontology (GO) and KEGG databases.

**Results:**

Seventy-six genes were differentially expressed in CKD muscle (FDR < 0.05, |log₂FC| ≥ 1), with 62 downregulated and 14 upregulated. he most consistent signature was suppression of immune-related and extracellular matrix transcripts, including CD163, C1QC, MPEG1, CXCL14, ITIH5, PODN, and CCDC80, suggesting attenuated immune surveillance and reduced ECM stability. In contrast, haemoglobin subunit genes (HBB, HBA1) were upregulated, potentially reflecting compensatory adaptation in oxygen transport. Several genes linked to regenerative processes (e.g., MEGF10, SOX4) were differentially expressed, but canonical myogenic and catabolic regulators remained unchanged, indicating that CKD muscle exists in a transcriptionally blunted state rather than one of overt inflammation or proteolysis.

**Conclusions:**

CKD skeletal muscle is characterised by suppression of immune and ECM regulatory programmes, with limited evidence for activation of classical inflammatory or degradative pathways. This distinct transcriptional profile suggests an immunologically and structurally quiescent state that may impair repair capacity and contribute to progressive sarcopenia. These findings refine current understanding of CKD-associated muscle dysfunction and highlight potential targets for mechanistic and therapeutic exploration.

## Introduction

Chronic kidney disease (CKD) is common, affecting around 14% of adults in England [1], for which there is no cure. A loss of muscle mass and strength (sarcopenia, which is now recognised as a disease in its own right [2]), is a frequent complication of CKD affecting around 28% of people with more advanced disease [3,4]. However, this starts early in the disease process [3] and results in a downward spiral of further muscle wasting, disuse, reduced quality of life and poor outcome [5]. Importantly, skeletal muscle is highly adaptive, easily remodelled through interventions such as exercise and nutrition, thus the sarcopenia that is observed in these patients is likely to be reversible or preventable. Any strategies that would be capable of improving muscle mass are an attractive means to improve quality of life, clinical outcomes and reduce healthcare costs. Unfortunately, treatments for sarcopenia in this patient group and others are lacking, which is primarily hampered by our lack of complete understanding of the processes that underlie this condition. Currently the most effective strategy to reduce or prevent sarcopenia is exercise [6]. Whilst effective, activity levels of CKD patients are low [7] and not all patients can or want to take part in exercise programmes. This is further compounded by the lack of formalised rehabilitation programmes available to CKD patients and therefore a lack of support exists to help patients to take part in regular exercise. This means we need to look to alternative strategies to help reduce/prevent sarcopenia in those patients not taking part in exercise. In order to design these alternative therapies for sarcopenia we must first understand in more detail the processes by which sarcopenia is initiated.

Multiple pathways and factors have been implicated in driving the loss of muscle mass in individuals with chronic kidney disease (CKD), including inflammation, oxidative stress, metabolic acidosis, insulin resistance, aberrant microRNA expression, and physical inactivity [8–12]. Microarray-based studies have previously examined skeletal muscle gene expression in this population and reported an enrichment of genes involved in inflammatory pathways [12]. However, high-throughput RNA sequencing (RNA-seq) exploring differential gene expression and underlying molecular pathways linked to sarcopenia in CKD remain limited. To address this, we performed RNA-seq on vastus lateralis muscle biopsies from 10 patients with CKD and 9 healthy controls to identify candidate pathways associated with muscle wasting.

In this study, we performed bulk RNA sequencing of vastus lateralis muscle biopsies from individuals with CKD and matched healthy controls to explore transcriptional mechanisms underlying CKD-associated sarcopenia. We identified a distinct transcriptomic profile characterised by suppression of immune-related and regenerative pathways, while classical markers of inflammation and protein degradation were largely unchanged. These findings suggest that CKD muscle exists in a blunted or quiescent transcriptional state, with impaired capacity for repair and adaptation. By refining our understanding of the molecular landscape of CKD muscle dysfunction, this work highlights potential biological targets for therapeutic strategies beyond conventional exercise-based interventions.

## Methods

### Participants and recruitment

CKD patients were recruited from nephrology outpatient clinicals at Leicester General Hospital, UK between December 2013 and January 2019, as part of the ExTra CKD trial (Ref/10/H0406/50). Healthy controls were recruited from the University of Leicester and University Hospitals of Leicester NHS Trust during hospital attendance for routine planned orthopaedic surgery as part of the Explore CKD trial and received ethical approval from the East midlands Leicester South Research Ethics Committee (15/EM/0467). Exclusion criteria included: age < 18 years, physical impairment sufficient to prevent undertaking exercise or exercise testing, recent myocardial infarction, active cancer, unstable chronic conditions, HbA1C<9% and an inability to give informed consent. Approval for all studies was granted from the UK National Research Ethics Committee. All participants gave written informed written consent and trials were conducted in accordance with the Declaration of Helsinki. The samples were accessed for research purposes between September 2019 and December 2023.

### Muscle biopsy sampling and processing

Biopsies were collected from the vastus lateralis muscle under fasted conditions. CKD participants donated a biopsy using the micro biopsy technique as previously described [13]. Healthy controls had a biopsy taken at the same time of a scheduled procedure for the removal of benign intramuscular lipomas [14] using the open biopsy technique. Following dissection of any visible fat, samples were immediately placed in liquid nitrogen and stored until subsequent analysis. Individual stored CKD samples were paired with a control sample matched for age (within two years), gender, ethnicity and physical activity levels assessed using the GP Physical Activity Questionnaire (GPPAQ).

### RNA extraction and transcriptome sequencing

RNA was extracted from 5 mg tissue using the miRNeasy micro kit according to the manufacturer’s instructions (Qiagen Inc, Valencia, CA). RNA quality was assessed using the Ailgent 5400 fragment analyzer system. All library preparation and subsequent RNA sequencing was performed by Novogene (Beijing, China) using poly(A)+ enrichment to capture polyadenylated transcripts, followed by sequencing on the Illumina Novoseq 6000 platform with a 150 bp paired-end strategy. HISAT2 was used to align the sequencing reads to the reference genome (Homo sapiens GRCG38/hg38).

### Statistical analysis

Gene level counts were generated from aligned reads using featureCounts (v2.01 [15]) to produce raw read counts. These counts were then supplied directly to DESeq2 (v1.38.0 [16]), which applies its internal median-of-ratios normalisation method to correct for sequencing depth and composition bias. No CPM, RPKM, or FPKM normalisation was applied prior to DESeq2. Quality filtering was performed prior to read alignment using Novogene’s standard pipeline (fastp v0.20.0 [17]). Reads with adapter contamination, reads when it was uncertain nucleotides constitute more than 10% of either read, and reads with low quality nucleotides that constitute of more than 10% of either read were removed. The reads were aligned to a reference genome (Homo Sapiens(GRCh38/hg38)) using HISAT2 (v2.2.1 [18]). To determine reliability of the analysis, correlation of the gene expression levels between samples was determined by Persons correlation. Principal component analysis (PCA) was used to evaluate intergroup differences and intragroup sample duplication. Due to the presence of biological replicates differential gene expression (DEG) analysis was performed using the DESeq2 package in R using the negative binomial distribution model using the design formula ~condition. No additional co-variates were included in the design. QQ plots were generated in R from the full set of tested genes using raw p-values (not FDR-adjusted). Hierarchical clustering analysis (HCA) and heatmap generation were performed using the pheatmap package in R (v1.0.12). PCA and volcano plots were generated using ggplot2 (v3.4.2). Functional enrichment analyses were conducted using clusterprofiler (v4.81 [19]). Genes with adjusted P value (FDR) <0.05 and |log2(FoldChange)|>=1 were considered differentially expressed, using the Benjamini-Hochberg procedure to control for false discovery rate [20]. A hierarchical clustering analysis of DEGs was performed using the ggplot2 package in R. Over representation analyses (ORA) (Gene Ontology (GO) [21] and Kyoto Encylopedia of Genes and Genomes (KEGG) pathway [22]) were performed on the 76 DEGs (FDR < 0.05; |log_2_FC| > 1. The primary background was restricted to genes detected in both groups (mean FPKM >1 in CKD and controls, n = 9765). GO terms and KEGG pathways with padj < 0.05 were deemed significantly enriched. As a sensitivity analysis, enrichment was repeated using the full set of genes that passed QC and were tested by DESqe2 (n = 14506).

## Results

### Participant characteristics

Of the planned matched pairs, one healthy control sample did not pass RNA quality control and a replacement was not available, resulting in 9 control and 10 CKD samples being included in the final analysis. Participant characteristics are shown in Table 1. As expected, the CKD group had substantially lower kidney function (median eGFR 24 vs. 86 mL/min/1.73m² in controls), consistent with stage 4 CKD. Despite this, groups were otherwise closely matched for age, sex, ethnicity, and physical activity levels, minimising potential confounding. Haemoglobin and albumin concentrations were modestly lower in CKD, reflecting the anaemia and metabolic milieu typical of advanced kidney disease. Functional capacity, assessed by the Sit-to-Stand 5 test, was impaired in the CKD group (median 10.5 s), supporting the presence of reduced muscle performance even in this relatively robust cohort. These baseline features provide the clinical context for the observed transcriptomic differences.

**Table 1:** Participant characteristics.

	CKD group (n = 10)	Healthy control group (n = 9)
**Number of Males (%)**	3 (30%)	3 (30%)
**Age (y)**	63 (27-78)	57 (29-75)
**eGFR (mL/min/1.73m^2^)**	24 (15-32)	86 (62-100)
**Haemoglobin (g/dL)**	117 (102-161)	137 (114-154)
**Albumin (g/L)**	41 (23-47)	38 (36-41)
**Comorbidities (n)**		
**Type II Diabetes**	0	0
**Essential hypertension**	3	0
**Ischaemic Heart Disease**	0	0
**Valvular heart disease**	1	0
**Sit-to-Stand 5 (secs)**	10.5 (3.6-14.0)	NR

Data are shown as median and range. Abbreviations: BMI, Body Mass Index; CKD, chronic kidney disease; eGFR, estimated glomerular filtration rate; NR, not recorded.

### RNA-seq quality assessment and sample clustering

RNA sequencing data were obtained from vastus lateralis muscle biopsies of 10 individuals with CKD and 9 healthy controls. After quality filtering and trimming, an average of ~25 million clean paired-end reads per sample were retained (range: 22.7–28.9 million), with a mean alignment rate of over 94% to the reference human genome. The sequencing quality was consistently high across all samples, with Q30 scores exceeding 94%, GC content around 51%, and error rates between 0.02% and 0.03%. These metrics indicate excellent sequencing depth and accuracy suitable for downstream analyses.

**Fig 1:**
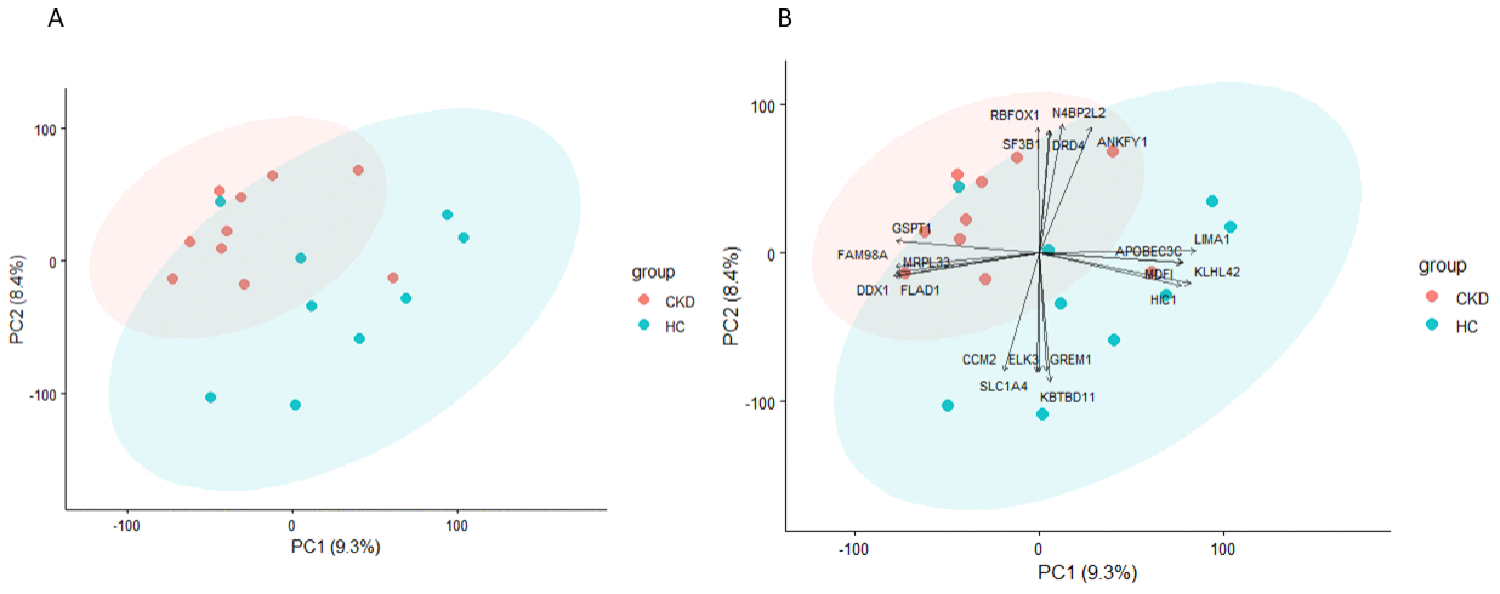
Principal component analysis of transcriptomic profiles in CKD and healthy control skeletal muscle. **(A)** PCA plot log₂(FPKM+1) displaying the first two principal components (PC1 and PC2) of gene expression profiles from vastus lateralis muscle biopsies of 10 individuals with chronic kidney disease (CKD; red circles) and 9 healthy controls (HC; green circles). **(B)** Biplot highlighting top gene loadings contributing to PC1 and PC2. Gene symbols are shown for transcripts with the strongest contributions to separation between groups.

Principal component analysis (PCA) was performed to assess sample-level variability and clustering based on global gene expression profiles. PC1 and PC2 accounted for 9.3% and 8.4% of the total variance, respectively (Fig 1). Despite the modest proportion of variance explained by these axes, samples clustered distinctly according to disease status, with CKD and control groups separated along PC1. One control sample showed greater variability and clustered closer to the CKD samples; however, all samples passed stringent RNA quality and sequencing QC thresholds. Differential expression analysis was performed using linear modelling approaches that are robust to within-group variability, and the overall results were unaffected by sensitivity analyses excluding this sample. Pearson correlation analysis of transcriptome-wide expression values showed uniformly high consistency across both intra-group (mean r = 0.938 for CKD, r = 0.930 for controls) and inter-group comparisons (r = 0.931), indicating strong reproducibility of the dataset. Inspection of PCA loadings provided further insight into the transcripts driving separation between groups (Fig 1B). Genes with the strongest contributions to PC1 and PC2 included ANKFY1, KLHL42, N4BP2L2, and CCM2.

### Gene signature of skeletal muscle from patients with CKD

**Fig 2:**
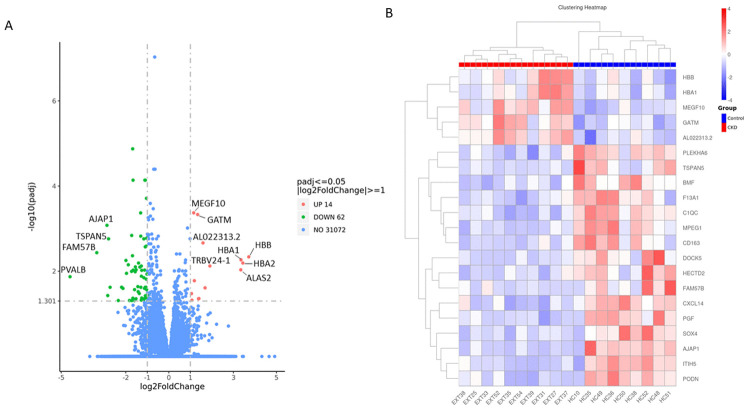
Differential gene expression between CKD and healthy control skeletal muscle. **(A)** Volcano plot showing differentially expressed genes (DEGs) between CKD and control muscle based on adjusted p-value < 0.05 and |log₂ fold change| ≥ 1. Upregulated genes in CKD are shown in red (n = 14), downregulated genes in blue (n = 62), and non-significant genes in grey. Selected top-ranking DEGs by significance are annotated. **(B)** Heatmap of the top 20 DEGs ranked by adjusted p-value. Z-score normalised expression values are shown across all samples (CKD, n = 10; controls, n = 9), with unsupervised hierarchical clustering applied by row. Red indicates higher relative expression and blue indicates lower relative expression.

On average 10,025 genes were found to be expressed (FPKM>1) in the CKD group and 10,232 genes in the healthy control group. A total of 9765 genes were shared between groups. Of these, 467 genes were uniquely expressed in the control group and 260 were unique to the CKD group. Differential gene expression analysis identified a total of 76 genes that were significantly altered in CKD muscle compared to controls (FDR < 0.05), with 14 genes upregulated and 62 downregulated (Fig 2A). The complete list of all expressed genes can be found in supplementary file 1 in S1 Data. The full RNA-seq data set is available at https://github.com/emmawatson0604/CKD-vs-Healthy-control-RNA-seq-data.

### Differential gene expression overview

**Fig 3:**
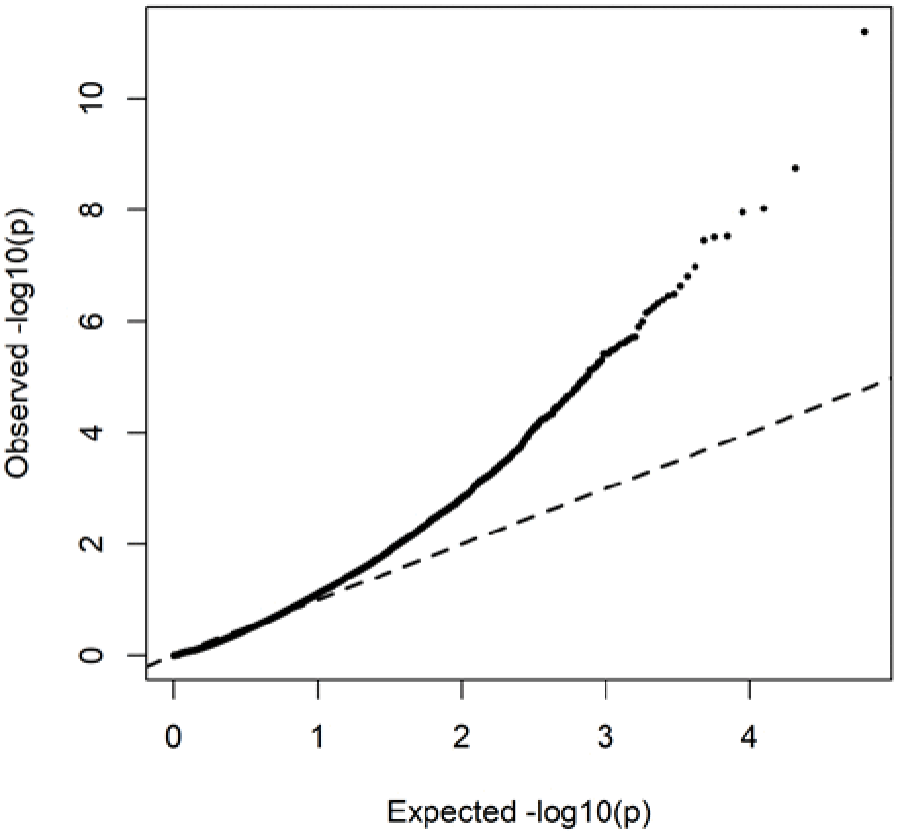
Quantile–quantile (QQ) plot of raw p-values from the CKD vs healthy control differential expression analysis (all tested genes). Observed −log10(p) values (points) are plotted against the expected −log10(p) under the null hypothesis (dashed line). p-values are unadjusted; entries with missing p-values were excluded. Generated in **R.**

To gain initial insight into potential functional mechanisms, we examined the top 20 DEG’s (up or down regulated), ranked by adjusted p-value (Table 2). Presenting the top 20 regulated genes provides a concise and interpretable overview. The full list of DEG’s identified by DESeq2 is available in Supplementary Table 2 in S2 File for completeness. Several of these genes, including AJAP1, C1QC, DOCK5, MPEG1, ITIH5, TSPAN5, PGF, FAM57B and BMF, have no well-characterised role in skeletal muscle biology. Others, however, have known or plausible involvement in processes relevant to muscle health and pathology. For example, CXCL14 [23], PODN [24], SOX4 [25] and MEGF10 [26] are linked to cell proliferation and differentiation, repair and regeneration; GATM [27] is involved in creatine biosynthesis; HBB and HBA1 are components of haemoglobin [28]; and F13A1 has been documented to have a role in insulin sensitivity [29]. These top-ranking DEGs highlight a combination of known and potentially novel contributors to CKD-associated muscle wasting. Notably, unsupervised clustering of these genes revealed a clear separation between CKD and control groups in the heatmap (Fig 2B), supporting a distinct transcriptomic profile in CKD muscle. To assess robustness, we repeated the analysis using a stricter threshold (|log₂FC| ≥ 1.5). This reduced the DEG set from 76 to 35, but key immune- and haemoglobin-related transcripts (CD163, C1QC, MPEG1, HBB, HBA1, ALAS2) remained significantly altered, and enrichment analyses continued to highlight immune surveillance and oxygen transport pathways. A full list of the DEGs identified at this stricter threshold is provided in Supplementary File 3 and Supplementary Fig 1 in S1 Data. The QQ plot of raw p-values (all tested genes) closely followed the expected null distribution across most quantiles, with deviation confined to the extreme right tail among the most significant tests (Fig 3)

**Table 2:** 20 most differentially expressed genes, up or down-regulated, based on P values together with logFC and adjusted P values. The comparison is healthy controls to the CKD cohort, negative fold changes indicate downregulation in CKD and positive values indicate up regulation in CKD.

Gene ID	Description	Log2FC	Adjusted P-value
HBB	hemoglobin subunit beta	3.7	0.004
HBA1	hemoglobin subunit alpha 1	3.3	0.005
GATM	glycine amidinotransferase	1.33	<0.001
MEGF10	multiple EGF like domains 10	1.15	<0.001
PLEKHA6	pleckstrin homology domain containing A6	−1.03	0.001
PODN	podocan	−1.04	<0.001
BMF	Bcl2 modifying factor	−1.05	0.002
CXCL14	C-X-C motif chemokine ligand 14	−1.09	<0.0001
HECTD2	HECT domain E3 ubiquitin protein ligase 2	−1.09	0.002
DOCK5	dedicator of cytokinesis 5	−1.1	0.001
ITHI5	inter-alpha-trypsin inhibitor heavy chain family member 5	−1.11	<0.0001
SOX4	SRY-box 4	−1.3	0.001
PGF	placental growth factor	−1.31	<0.001
F13A1	coagulation factor XIII A chain	−1.6	0.004
MPEG1	Macrophage expressed 1	−1.66	<0.0001
C1QC	protein tyrosine phosphatase, receptor type G	−1.68	<0.0001
CD163	CD163 molecule	−1.7	0.001
TSPAN5	tetraspanin 5	−2.8	0.001
AJAP1	adherens junctions associated protein 1	−2.87	<0.001
FAM57B	family with sequence similarity 57 member B	−3.3	0.003

### Functional enrichment analyses

**Fig 4:**
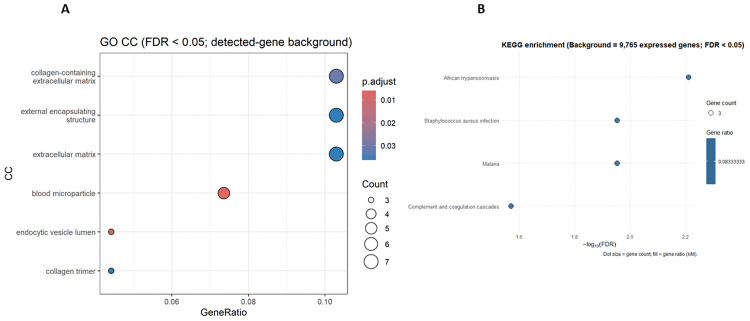
Functional enrichment analysis of differentially expressed genes in CKD skeletal muscle. **(A)** Gene Ontology (GO) enrichment analysis of 76 DEGs identified in CKD versus healthy control muscle (adjusted p < 0.05, |log₂ fold change| ≥ 1). **(B)** KEGG pathway enrichment analysis of 76 DEGs identified in CKD versus healthy control muscle (adjusted p < 0.05, |log₂ fold change| ≥ 1).

To understand the function implications of all DEGs we carried out GO pathway enrichment and KEGG analyses on the 76 DEGs to further explore the biological processes dysregulated in CKD skeletal muscle (Fig 4). Because ORA is sensitive to background selection, we used a detected-gene background (genes expressed in both groups; n = 9,765) for our primary analysis. Under these conditions, GO biological process and molecular function did not yield any terms passing FDR < 0.05. In contrast, GO cellular component identified six significant terms, including blood microparticle (5/68), collagen trimer (3/68), external encapsulating structure (7/68) endocytic vesicle lumen (3/68), extracellular matrix (7/68) and collagen-containing extracellular matrix term (7/68 – that included C1QC, ITIH5, PODN, C1QA, NTN4, CCDC80 and C1QB, all of which were downregulated in the CKD cohort. This may be consistent with dysregulated extracellular matrix components. The blood microparticle/vesicle lumen terms align with the observed haemoglobin upregulation (HBB, HBA1, HBA2). To complement the GO analysis, we performed KEGG over-representation testing. When restricting the background to the 9,765 genes detected across groups, few pathways survived multiple-testing correction (FDR < 0.05) Complete outputs for both backgrounds (all terms and significant-only subsets) are provided as Supplementary files 8–11 in S1 Data.

As a complementary analysis, we also performed Gene Set Enrichment Analysis (GSEA) on the full ranked gene list using both Gene Ontology and KEGG databases. This approach did not identify any pathways reaching statistical significance at the conventional FDR < 0.25 threshold. The complete results are provided in Supplementary File 12 in S1 Data.

### Functional enrichment sensitivity analysis

When we repeated ORA using the full DESeq2-tested background (n = 14,506 genes), GO–BP yielded 7 FDR-significant terms (FDR range 0.024–0.043), including humoral immune response (6/68), complement activation (4/68), and one-carbon compound transport (3/68). GO–CC returned 6 terms (FDR 0.0005–0.044), led by blood microparticle (6/68), extracellular matrix*/*external encapsulating structure (10/68 each), and collagen-containing extracellular matrix (9/68). GO–MF identified 9 terms (FDR 0.001–0.048), including cargo receptor activity (5/69), heme binding and tetrapyrrole binding (4/69 each), plus opsonin binding *and* complement binding. These patterns align with the gene-level signals—upregulated haemoglobin transcripts consistent with heme/tetrapyrrole binding and blood microparticle terms, and downregulated immune/complement components (e.g., CD163, C1QA/C1QC, MPEG1, CXCL14) consistent with humoral/complement processes, while highlighting that statistical significance depends on background choice. (See Supplementary Fig 2A–C and Supplementary files 4–7 in S1 Data for full term lists and statistics.)

### Suppressed immune-related signaling in CKD muscle and an upregulation of oxygen transport capacity

To understand the function implications of all DEGs we carried out GO pathway enrichment and KEGG analyses on the 76 DEGs to further explore the biological processes dysregulated in CKD skeletal muscle. Using the detected-gene background (n = 9765), no GO Biological Process terms reached FDR < 0.05. Nevertheless, the gene-level pattern showed downregulation of immune-associated transcripts (e.g., CD163, C1QC, MPEG1, CXCL14) and upregulation of haemoglobin genes (HBB, HBA1). In a sensitivity analysis using the full DESeq2-tested universe (n = 14,506 genes), immune processes such as humoral immune response, immunoglobulin-mediated immunity and complement activation reached FDR < 0.05 (Supplementary Fig 2 in S1 Data; Supplementary Table 7 in S7 File), consistent with the gene-level signal.”

In support of this, KEGG analysis revealed several significantly enriched pathways, many of which align with the immune-related signals observed in the GO results. These included systemic lupus erythematosus*,* Staphylococcus aureus infection*,* and tuberculosis, pathways, commonly enriched in transcriptomic analyses due to the presence of genes related to immune surveillance, complement activation, and haemoglobin metabolism. These were primarily driven by downregulation of HBB, HBA1, CXCL14, C1QA, C1CB and CD163, reinforcing the finding of attenuated immune and redox signalling in CKD muscle (Fig 3B). Full KEGG results are presented in supplementary file 8 in S1 Data. To avoid over-interpretation, we do not ascribe disease specificity to this pattern, as similar patterns have been found in other chronic diseases [30,31]. Rather, our data indicate an attenuated immune–repair transcriptional programme in CKD muscle, which is hypothesis-generating pending and require much further validation.

### Evidence of impaired regeneration and repair signatures

Although global enrichment analysis did not strongly highlight classical regeneration or muscle repair pathways, closer inspection of the top-ranking DEGs (Table 2) revealed transcriptional changes in several genes with established or plausible roles in these processes. MEGF10, required for satellite cell function and muscle regeneration [26], was among the most significantly downregulated genes in CKD muscle. Similarly, the macrophage scavenger receptor, CD163, important for tissue repair and resolution of inflammation [32], and C1QC, a complement component essential for clearance of apoptotic cells during regeneration, were both downregulated [33]. In addition, the chemokine CXCL14 was reduced in CKD; CXCL14 regulates immune cell recruitment and its upregulation has been implicated in muscle regeneration and metabolic regulation [23]. Genes implicated in extracellular matrix organisation and repair were also supressed, including PODN, a small leucine-rich repeat proteoglycan, with reported roles in ECM assembly and skeletal muscle repair [34]. Taken together, these changes suggest the attenuation of regenerative and reparative signalling in CKD muscle. However, expression of canonical myogenic regulators such as MyoD and Myogenin was unchanged.

Taken together, these findings indicate that CKD may subtly impair key components of the muscle’s intrinsic regenerative and metabolic capacity. Although not dominant at the pathway level, these gene-level changes may contribute to the progressive decline in muscle quality and resilience observed in individuals with chronic kidney disease.

## Discussion

This study reports a distinct transcriptomic profile of skeletal muscle in patients CKD, revealing significant downregulation of immune and extracellular matrix (ECM) pathways. Several innate immune transcripts with established roles in tissue surveillance and repair, including CD163, C1QC, MPEG1, and CXCL14, were markedly downregulated, alongside reduced expression of ECM components such as C1QA/B/C, ITIH5, PODN, CCDC80, and NTN4, which are critical for maintaining structural stability and immune–matrix interactions. These changes suggest an attenuated immune–ECM programme, which may compromise the muscle’s ability to sustain homeostasis and respond to injury. In contrast, haemoglobin subunit genes (HBB, HBA1) were upregulated, potentially reflecting a compensatory adaptation in oxygen handling. alongside a small number of potentially compensatory upregulated genes.

One of the most prominent themes emerging from our data is the downregulation of immune-related genes and pathways in CKD skeletal muscle. Gene Ontology and KEGG enrichment analyses indicated suppression of leukocyte chemotaxis and cytokine receptor activity, alongside enrichment terms populated by broadly reduced innate-immune transcripts that also have reparative or homeostatic roles. In context, immune cells are key coordinators of muscle maintenance and regeneration, macrophages transition from pro-inflammatory to pro-regenerative states to clear debris and support myogenesis, while regulatory T cells modulate these phases to enable effective repair [35]. Disruption of these programs is linked to impaired regeneration with age [36], and furthermore, inflammation is a well-defined driver of age-related muscle loss. [37] In parallel, systemic inflammation is a recognised contributor to muscle wasting in CKD, where cytokine signalling promotes proteolysis and interferes with myogenesis [38]. Consistent with an immune component to sarcopenia more broadly, higher circulating inflammatory markers (e.g., IL-6, CRP) are associated with lower muscle mass/strength and physical performance in CKD [8].

Within our DE gene set, CD163, an anti-inflammatory scavenger receptor on M2-like macrophages—was significantly downregulated. CD163 typically increases with inflammatory cues (including glucocorticoids/IL-10) and its soluble form is elevated across inflammatory conditions, highlighting its role in macrophage-mediated resolution [39] By contrast, the present RNA-seq showed no increase in classic cytokines (IL6, TNF, CCL2), differing from our prior qPCR report in a related cohort. [8] We interpret this as a combination of biological heterogeneity (distinct individuals sampled), methodological differences (targeted qPCR versus genome-wide RNA-seq with multiple-testing correction), and possible cell-composition effects in bulk tissue, rather than a contradiction of immune involvement. Additional downregulated transcripts reinforce this picture: MPEG1 (macrophage-expressed gene 1), an innate antimicrobial effector central to phagocyte defence; DOCK-family Rho-GEFs (e.g., DOCK5/DOCK10) that participate in leukocyte signalling and migration [40] and CXCL14, a chemokine implicated in immune-cell recruitment and tissue homeostasis [41]. Interesting one previous study has shown CXCL14 depletion has been shown to enhance myogenesis by promoting cell cycle withdrawl. [23] A reduction in CXCL14 here could be interpretated as a compensatory mechanism to facilitate muscle repair, this warrants further investigation.

A reduced expression of CD163 may also reflect a reduced presence of resident macrophages or a blunted repair response in the CKD muscle environment. Supporting this, other immune-associated genes, including MPEG1 and CXCL14 (a chemokine involved in immune cell recruitment and regeneration), were also significantly downregulated. The downregulation of DOCK5 and DOCK10, which regulate leukocyte signalling and migration, further reinforces a general attenuation of immune responsiveness. This attenuated immune transcriptional profile may suggest that CKD muscle may exist in a transcriptionally suppressed or immunologically quiescent state, impairing the muscle’s capacity to resolve damage and support regeneration in the CKD environment. Whether this immune–repair signature is CKD-specific versus a shared feature of chronic disease remains unresolved; definitive attribution will require cross-condition analyses and functional validation in appropriate cellular and in vivo models”

In addition to the suppression of immune-related signalling, several extracellular matrix (ECM)-associated transcripts were also significantly downregulated in CKD muscle, including C1QA, C1QB, C1QC, ITIH5, PODN, CCDC80, and NTN4. These proteins are known to contribute to diverse aspects of ECM homeostasis, ranging from immune–matrix crosstalk (C1q complex) and matrix stabilisation (ITIH5) to regulation of fibrosis (PODN, CCDC80) and vascular support (NTN4). Reduced expression of these genes may therefore reflect a loss of protective regulatory mechanisms that normally maintain ECM balance and restrain maladaptive structural remodelling. Taken together with the broader suppression of immune signalling, these findings suggest that CKD muscle exists in an environment less able to support tissue repair and more susceptible to progressive structural decline.

In addition to this, our data also provide further support for impaired muscle regeneration and repair capacity in CKD. Podocan, which has been shown to have a role in muscle regeneration [34] was down regulated in CKD, together with SOX4, that has been shown to facilitate muscle myoblast differentiation [25]. Many of the DEGs with known roles in myogenesis and satellite cell activation were either downregulated or unchanged in CKD muscle compared to healthy controls. Key canonical markers of satellite cell activation and differentiation, including PAX7*,* MYOD1*, and* MYOGENIN*,* were not differentially expressed, together with down regulation of PODN, a repair-associated gene.

Interestingly*,* MEGF10, which plays a critical role in satellite cell-mediated muscle regeneration [26], was significantly upregulated in CKD muscle. MEGF10 knockout mice have reduced muscle mass and neuromuscular junction instability [42] suggesting an important role in maintenance of muscle health. This may represent a compensatory mechanism to preserve the regenerative potential of muscle in response to chronic muscle stress. We also reported downregulation of CXCL14. A recent study has shown that reduced CXCL14 expression may facilitate satellite cell activation and promote regeneration, especially in injury contexts [23]. Thus, the suppressed expression of *CXCL14* in CKD muscle may reflect an adaptive, pro-myogenic shift.

This regenerative profile likely reflects the resting, non-injured state of the individuals at the time the muscle biopsies were collected. This profile is difficult to interpret with lack of a stimulus that would stimulate these pathways. Our previous data has suggested that in an exercise naïve individual combined resistance and aerobic exercise is unable to activate processes of myogenesis, a process that was restored following training, suggesting impaired regenerative responses [43].

Interestingly, we did not detect any change in expression of key drivers of protein degradation such as myostatin, MuRF-1 or MAFbx, in contrary to our previous report [8], suggesting there was no overt activation of skeletal muscle protein degradation under basal conditions. This discrepancy likely reflects both methodological factors (sensitivity of targeted qPCR versus genome-wide RNA-seq with multiple testing correction) and, importantly, the use of different biopsy cohorts. Even within the same patient population, skeletal muscle samples can exhibit substantial inter-individual and intra-muscle variability. Nevertheless, despite differences at the single-gene level, both studies converge on a consistent biological theme, highlighting dysregulation of immune signalling in CKD muscle.

A limitation of this study is the small cohort size, which may reduce the statistical power to detect genes influenced by CKD by small fold changes. To maintain a conservative approach, we applied a stringent threshold (adjusted p-value < 0.05 and |log₂FC| ≥ 1) to define differentially expressed genes. While this enhances specificity, it may also exclude genes with more modest changes that contribute to disease pathology, particularly in a complex and multifactorial condition like CKD. We also performed Gene Set Enrichment Analysis (GSEA) using the full ranked gene list to provide complementary pathway-level context. This analysis did not identify any pathways reaching statistical significance at FDR < 0.25, likely reflecting the relatively small sample size, the modest fold changes observed, and the conservative statistical thresholds applied. A limitation of the analysis specifically is that differential expression was modelled only as a function of disease status and no other co-variates were used. While DESeq2’s internal normalisation corrects for sequencing depth, other potential covariates such as RNA integrity, age or sex were not explicitly included in the design. Although participants were closely matched on key demographic factors (age, gender, ethnicity and physical activity levels), residual confounding cannot be excluded. The enrichment analysis was based on a relatively small number of DEG’s (n = 76), which increases the potential for false-positives. One counts were generated from aligned reads using featureCounts the analysis and contributed to the apparent enrichment of coagulation cascade pathways. However, all samples were carefully dissected to remove visible fat and non-muscle tissue, and the sampling procedure was consistent across groups. Furthermore, the DEG profile was not restricted to haemoglobin-related genes, but also encompassed immune and regenerative markers, suggesting the findings more likely reflect intrinsic alterations in skeletal muscle biology. It should be noted that the CKD cohort in this study was relatively robust, with STS5 performance well below the EWGSOP2 threshold for probable sarcopenia [44]. This likely reflects the selection of participants from an exercise training study, and indicates that the transcriptional suppression of immune and regenerative pathways we observed may emerge even before the clinical onset of sarcopenia. The prevalence of sarcopenia increases markedly with advancing kidney disease, with up to 64% of haemodialysis patients classified as having probable sarcopenia [45]. Our findings may therefore represent early transcriptomic alterations that intensify with disease progression. Future analyses of skeletal muscle from more severely affected patients, such as those on dialysis, will be an important comparison.

The use of bulk RNA sequencing does not resolve gene expression at the level of specific cell types. Given the cellular heterogeneity of skeletal muscle—including myofibres, immune cells, endothelial cells, and fibro-adipogenic progenitors—some transcriptional changes may reflect shifts in cell composition rather than regulation within a specific cell type. Future studies incorporating single-cell or spatial transcriptomics could help disentangle these effects. Finally, transcriptomic data alone cannot provide insight into post-transcriptional regulation or protein-level effects. Some differentially expressed genes may not translate into functional changes, and integration with proteomics or histological data would be valuable to corroborate key findings.

In conclusion, we identified a distinct gene expression signature in CKD muscle characterised by suppression of immune pathways, alongside potential compensatory upregulation of genes involved in oxygen transport. Notably, classical inflammatory mediators and myogenic regulators were either unchanged or downregulated, suggesting that CKD muscle may exist in a blunted or unresponsive state rather than a persistently inflamed one. In addition to suppressed immune signalling, our data reveal downregulation of key extracellular matrix regulators, suggesting that early disruption of ECM homeostasis may contribute to CKD-associated muscle dysfunction. This loss of protective ECM components could create a permissive environment for maladaptive remodelling, further compounding impaired repair capacity. These findings refine our understanding of the molecular landscape in CKD-associated muscle dysfunction and highlight potential targets for future mechanistic and therapeutic exploration. Larger, longitudinal, and functionally validated studies will be required to further elucidate the drivers and reversibility of these transcriptional alterations.

## Supporting information

S1 Data S1 File. Complete list of differentially expressed genes (DEGs) between CKD and control skeletal muscle identified by DESeq2. Columns include Ensembl gene ID, gene symbol, log₂ fold change (CKD vs control), nominal p-value, adjusted p-value (Benjamini–Hochberg FDR), and gene description. Genes are ranked by adjusted p-value. A threshold of adjusted p-value (FDR) < 0.05 and |log₂ fold change| ≥ 1 was applied to define significance. S2 File. Significant differentially expressed genes (DEGs) between CKD and control skeletal muscle. Genes were identified using DESeq2 with adjusted p-value (FDR) < 0.05 and |log₂ fold change| ≥ 1. The table includes Ensembl gene ID, gene symbol, log₂ fold change (CKD vs control), nominal p-value, adjusted p-value (Benjamini–Hochberg FDR), and gene description. These 76 genes represent the subset of the full DESeq2 output (see Supplementary Table 1 in S1 File) that met statistical significance criteria and are highlighted in the manuscript Results. S3 File. Differentially expressed genes identified using a stricter threshold (|log₂FC| ≥ 1.5, adjusted p < 0.05). This table lists the 35 DEGs that remain significant when applying a more stringent fold-change cut-off. Columns include gene symbol, log₂ fold change (negative values indicate downregulation in CKD, positive values upregulation), and adjusted p-value (Benjamini–Hochberg). S4 File. Complete Gene Ontology (GO) enrichment analysis results using the 9,765-gene background. Enrichment was performed using clusterProfiler (v4.8.1) on the set of differentially expressed genes (DEGs) with adjusted p-value (FDR) < 0.05 and |log₂ fold change| ≥ 1, against a background of all expressed genes (n = 9,765). The table includes GO term ID, term description, gene set size, number of DEGs in the set, enrichment score, nominal p-value, adjusted p-value (Benjamini–Hochberg FDR), and the list of contributing genes. Both significant and non-significant GO terms are reported for transparency; terms with FDR < 0.05 are considered significantly enriched. S5 File. Functional enrichment analysis of differentially expressed genes (DEGs) in CKD skeletal muscle using the 9,765-gene background. Enrichment analysis was performed with clusterProfiler (v4.8.1), restricting the background to all 9,765 genes that passed QC and were tested for differential expression. Genes with an adjusted P value < 0.05 were considered significant. Only significantly enriched biological processes are shown. S6 File. Functional enrichment analysis of differentially expressed genes (DEGs) in CKD skeletal muscle using the full gene universe as the background. Enrichment analysis was performed with clusterProfiler (v4.8.1), using the default background of all annotated genes in the genome. Both significant and non-significant terms are shown to provide a complete overview of the analysis. S7 File. Significantly enriched biological processes among differentially expressed genes (DEGs) in CKD skeletal muscle using the full gene universe as the background. Enrichment analysis was conducted with clusterProfiler (v4.8.1), using the full genome as the background set. Only terms with an adjusted P value < 0.05 are shown. S8 File. Complete Kyoto Encyclopedia of Genes and Genomes (KEGG) pathway enrichment analysis results. Enrichment was performed using clusterProfiler (v4.8.1) on the set of differentially expressed genes (DEGs) with adjusted p-value (FDR) < 0.05 and |log₂ fold change| ≥ 1, against a background of all expressed genes (n = 9,765). The table includes KEGG pathway ID, pathway description, gene set size, number of DEGs in the set, enrichment score, nominal p-value, adjusted p-value (Benjamini–Hochberg FDR), and the list of contributing genes. Both significant and non-significant pathways are reported for transparency; pathways with FDR < 0.05 are considered significantly enriched. S9 File. Complete Kyoto Encyclopedia of Genes and Genomes (KEGG) pathway enrichment analysis results, significant terms only. Enrichment was performed using clusterProfiler (v4.8.1) on the set of differentially expressed genes (DEGs) with adjusted p-value (FDR) < 0.05 and |log₂ fold change| ≥ 1, against a background of all expressed genes (n = 9,765). The table includes KEGG pathway ID, pathway description, gene set size, number of DEGs in the set, enrichment score, nominal p-value, adjusted p-value (Benjamini–Hochberg FDR), and the list of contributing genes. Only pathways with FDR < 0.05 are reported. S10 File. Complete Kyoto Encyclopedia of Genes and Genomes (KEGG) pathway enrichment analysis results. KEGG over-representation analysis of DEGs using the full KEGG gene universe as background. Enrichment was performed using clusterProfiler (v4.8.1) on the set of differentially expressed genes (DEGs) with adjusted p-value (FDR) < 0.05 and |log₂ fold change| ≥ 1. The table includes KEGG pathway ID, pathway description, gene set size, number of DEGs in the set, enrichment score, nominal p-value, adjusted p-value (Benjamini–Hochberg FDR), and the list of contributing genes. Both significant and non-significant pathways are reported for transparency; pathways with FDR < 0.05 are considered significantly enriched. S11 File. Complete Kyoto Encyclopedia of Genes and Genomes (KEGG) pathway enrichment analysis results (full universe as background). KEGG over-representation analysis of DEGs using the full KEGG gene universe as background. Enrichment was performed using clusterProfiler (v4.8.1) on the set of differentially expressed genes (DEGs) with adjusted p-value (FDR) < 0.05 and |log₂ fold change| ≥ 1. The table includes KEGG pathway ID, pathway description, gene set size, number of DEGs in the set, enrichment score, nominal p-value, adjusted p-value (Benjamini–Hochberg FDR), and the list of contributing genes. Only pathways with FDR < 0.05 are reported. S12 File. Complete Gene Set Enrichment Analysis (GSEA) results. GSEA was performed using clusterProfiler (v4.8.1) on the full ranked list of expressed genes (n = 9,765), ranked by log₂ fold change (CKD vs control). Analyses were run against Gene Ontology (GO) and Kyoto Encyclopedia of Genes and Genomes (KEGG) databases. Results are presented separately for pathways enriched at the CKD end and the Control end of the ranked list. Each sheet provides the pathway name, normalized enrichment score (NES), nominal p-value, and FDR q-value. S1 Fig. Volcano plot showing differentially expressed genes (DEGs) between CKD and control muscle (adjusted p-value < 0.05 and |log₂ fold change| ≥ 1.5) repeated for robustness. Upregulated genes in CKD are shown in red (n = 28), downregulated genes in green (n = 7), and non-significant genes in blue. Selected top-ranking DEGs by significance are annotated. S2 Fig. GO enrichment of DEGs using the full tested background (sensitivity analysis). Over-representation analysis of the 76 DEGs (FDR < 0.05; |log₂FC| ≥ 1) with the full DESeq2-tested universe as background (n = 14,506 genes). Analyses were performed in R (clusterProfiler with Benjamini–Hochberg correction). Only terms with FDR < 0.05 are shown. (A) Biological Process (BP): GO BP terms significantly enriched among DEGs. (B) Cellular Component (CC): GO CC terms significantly enriched among DEGs. (C) Molecular Function (MF): GO MF terms significantly enriched among DEGs. In all panels, dot size denotes the number of DEGs in the term (Count), the x-axis shows the GeneRatio (k/76), and colour encodes the BH-adjusted FDR (red = more significant). (Primary analyses using the detected-gene background are presented in the main text.).
(ZIP)
