# Supplementary figures and images for: Transcriptomic suppression of immune and ECM stability in skeletal muscle of patients with chronic kidney disease
Source: PLoS One. 2026 Feb 24;21(2):e0328947. doi: 10.1371/journal.pone.0328947 (PMC12931797; doi:10.1371/journal.pone.0328947)

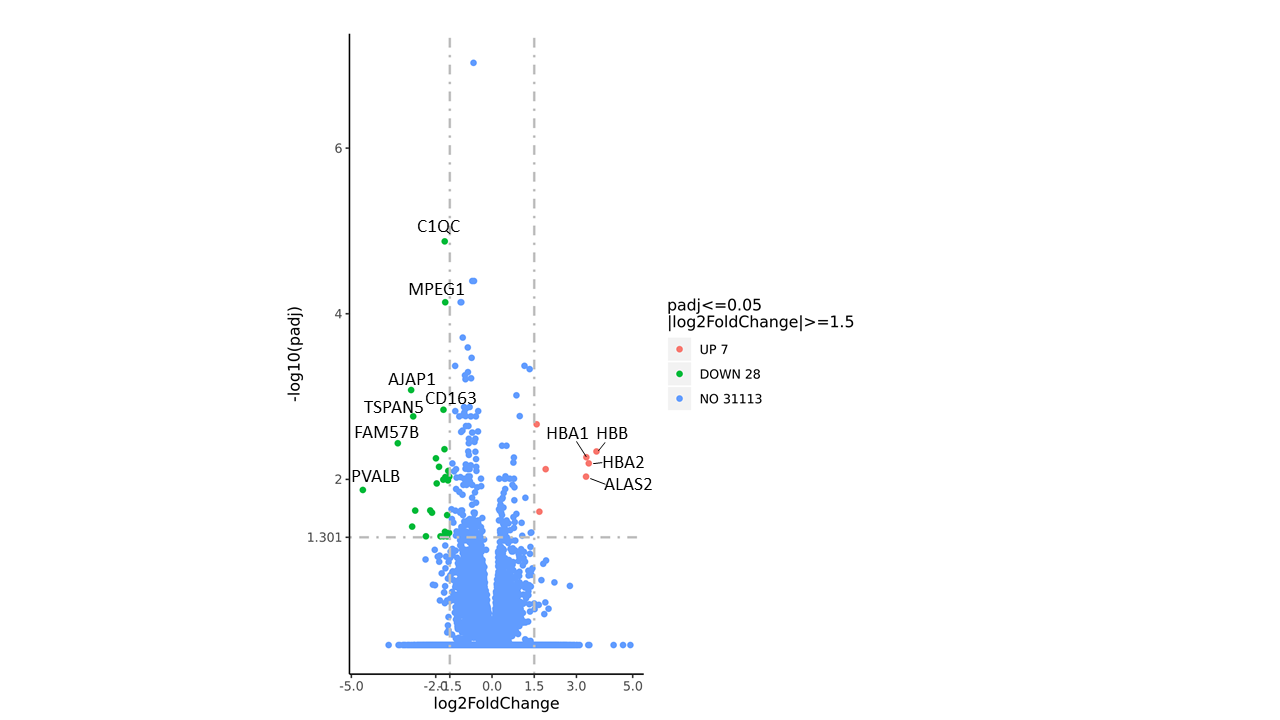

Supplement: S1 Data — S1 File. Complete list of differentially expressed genes (DEGs) between CKD and control skeletal muscle identified by DESeq2. Columns include Ensembl gene ID, gene symbol, log₂ fold change (CKD vs control), nominal p-value, adjusted p-value (Benjamini–Hochberg FDR), and gene description. Genes are ranked by adjusted p-value. A threshold of adjusted p-value (FDR) < 0.05 and |log₂ fold change| ≥ 1 was applied to define significance. S2 File. Significant differentially expressed genes (DEGs) between CKD and control skeletal muscle. Genes were identified using DESeq2 with adjusted p-value (FDR) < 0.05 and |log₂ fold change| ≥ 1. The table includes Ensembl gene ID, gene symbol, log₂ fold change (CKD vs control), nominal p-value, adjusted p-value (Benjamini–Hochberg FDR), and gene description. These 76 genes represent the subset of the full DESeq2 output (see Supplementary Table 1 in S1 File) that met statistical significance criteria and are highlighted in the manuscript Results. S3 File. Differentially expressed genes identified using a stricter threshold (|log₂FC| ≥ 1.5, adjusted p < 0.05). This table lists the 35 DEGs that remain significant when applying a more stringent fold-change cut-off. Columns include gene symbol, log₂ fold change (negative values indicate downregulation in CKD, positive values upregulation), and adjusted p-value (Benjamini–Hochberg). S4 File. Complete Gene Ontology (GO) enrichment analysis results using the 9,765-gene background. Enrichment was performed using clusterProfiler (v4.8.1) on the set of differentially expressed genes (DEGs) with adjusted p-value (FDR) < 0.05 and |log₂ fold change| ≥ 1, against a background of all expressed genes (n = 9,765). The table includes GO term ID, term description, gene set size, number of DEGs in the set, enrichment score, nominal p-value, adjusted p-value (Benjamini–Hochberg FDR), and the list of contributing genes. Both significant and non-significant GO terms are reported for transparency; terms with FD [file pone.0328947.s001.zip › S1 Fig.tif]

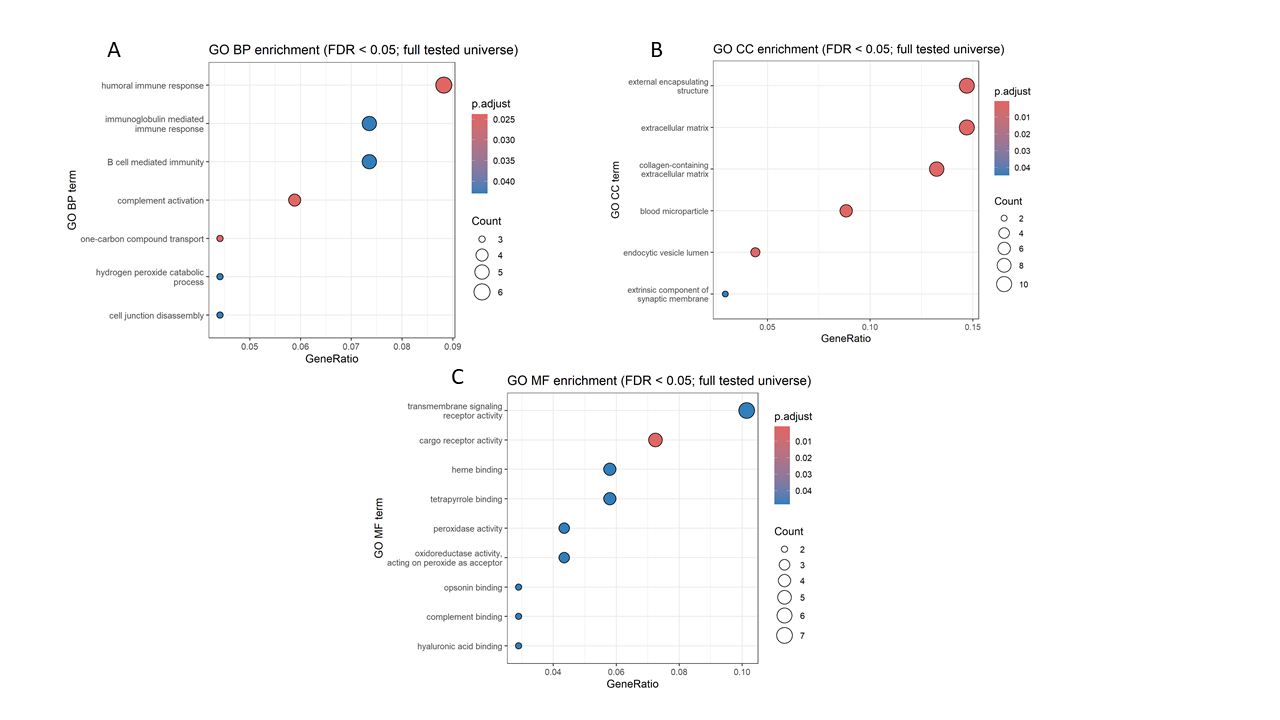

Supplement: S1 Data — S1 File. Complete list of differentially expressed genes (DEGs) between CKD and control skeletal muscle identified by DESeq2. Columns include Ensembl gene ID, gene symbol, log₂ fold change (CKD vs control), nominal p-value, adjusted p-value (Benjamini–Hochberg FDR), and gene description. Genes are ranked by adjusted p-value. A threshold of adjusted p-value (FDR) < 0.05 and |log₂ fold change| ≥ 1 was applied to define significance. S2 File. Significant differentially expressed genes (DEGs) between CKD and control skeletal muscle. Genes were identified using DESeq2 with adjusted p-value (FDR) < 0.05 and |log₂ fold change| ≥ 1. The table includes Ensembl gene ID, gene symbol, log₂ fold change (CKD vs control), nominal p-value, adjusted p-value (Benjamini–Hochberg FDR), and gene description. These 76 genes represent the subset of the full DESeq2 output (see Supplementary Table 1 in S1 File) that met statistical significance criteria and are highlighted in the manuscript Results. S3 File. Differentially expressed genes identified using a stricter threshold (|log₂FC| ≥ 1.5, adjusted p < 0.05). This table lists the 35 DEGs that remain significant when applying a more stringent fold-change cut-off. Columns include gene symbol, log₂ fold change (negative values indicate downregulation in CKD, positive values upregulation), and adjusted p-value (Benjamini–Hochberg). S4 File. Complete Gene Ontology (GO) enrichment analysis results using the 9,765-gene background. Enrichment was performed using clusterProfiler (v4.8.1) on the set of differentially expressed genes (DEGs) with adjusted p-value (FDR) < 0.05 and |log₂ fold change| ≥ 1, against a background of all expressed genes (n = 9,765). The table includes GO term ID, term description, gene set size, number of DEGs in the set, enrichment score, nominal p-value, adjusted p-value (Benjamini–Hochberg FDR), and the list of contributing genes. Both significant and non-significant GO terms are reported for transparency; terms with FD [file pone.0328947.s001.zip › S2 Fig.tif]
